# Mechanochemical Activation of Basic Oxygen Furnace Slag: Insights into Particle Modification, Hydration Behavior, and Microstructural Development

**DOI:** 10.3390/ma18153687

**Published:** 2025-08-06

**Authors:** Maochun Xu, Liuchao Guo, Junshan Wen, Xiaodong Hu, Lei Wang, Liwu Mo

**Affiliations:** 1 College of Materials Science and Engineering, Nanjing Tech University, Nanjing 211800, China; xumaochun_28@126.com (M.X.); guoliuchao@126.com (L.G.); leiwangdream@163.com (L.W.); 2 Jiangsu Province Hydraulic Research Institute, Nanjing 210017, China; xmc_njt@163.com; 3 China Railway Qinghai Tibet Group Co., Ltd., Xining 810007, China; secretfang66@163.com

**Keywords:** basic oxygen furnace slag, grinding, hydration, microstructural

## Abstract

This study proposed a mechanochemical activation strategy using ethanol-diisopropanolamine (EDIPA) to improve the grindability and hydration reactivity of basic oxygen furnace slag (BOFS), aiming for its large-scale industrial utilization. The incorporation of EDIPA significantly refined the particle size distribution and reduced the repose angle. As a result, the compressive strength of BOFS paste increased by 25.4 MPa at 28 d with only 0.08 wt.% EDIPA. Conductivity tests demonstrated that EDIPA strongly complexes with Ca^2+^, Al^3+^, and Fe^3+^, facilitating the dissolution of active mineral phases, such as C_12_A_7_ and C_2_F, and accelerating hydration reactions. XRD and TG analyses confirmed that the incorporation of EDIPA facilitated the formation of Mc (C_4_(A,F)ČH_11_) and increased the content of C-S-H, both of which contributed to microstructural densification. Microstructural observations further revealed that EDIPA refined Ca(OH)_2_ crystals, increasing their specific surface area from 4.7 m^2^/g to 35.2 m^2^/g. The combined effect of crystal refinement and enhanced hydration product formation resulted in reduced porosity and improved mechanical properties. Overall, the results demonstrated that EDIPA provided an economical, effective, and scalable means of activating BOFS, thereby promoting its high-value utilization in low-carbon construction materials.

## 1. Introduction

Basic oxygen furnace slag (BOFS) is the principal by-product of the steel-making process, accounting for approximately 15–20 wt% of crude steel production [1]. According to data from the National Bureau of Statistics of China, the cumulative stockpile of BOFS has surpassed one billion tons, while the utilization rate remains below 30%. This excessive accumulation not only occupies arable land but also poses serious environmental risks [2,3,4,5]. The limited use of BOFS in engineering applications is largely attributed to its poor cementitious activity, which stems from the presence of inert mineral phases (such as γ-C_2_S and RO phases) [6,7,8,9] and unstable components (such as f-CaO and f-MgO) [10,11]. In addition, its low grindability results in high energy consumption during fine grinding, further restricting its industrial application.

To improve the early hydration activity of BOFS, several methods have been investigated, including mineral phase reconstruction [12], chemical modification [13], steam curing [14], ultra-fine grinding [15,16] and carbonation [17,18,19,20]. However, these approaches suffer from various limitations such as strict requirements for compositional stability, high operational costs, and intensive energy consumption [21,22]. Although carbonation has shown potential in both stabilizing BOFS and reducing CO_2_ emissions, it demands stringent process control. Therefore, the development of a simple, efficient, and low-cost chemical activator is crucial for enhancing the reactivity and utilization of BOFS.

Alkanolamines, such as triethanolamine (TEA), triisopropanolamine (TIPA), diethanolisopropanolamine (DEIPA), and methyldiethanolamine (MDEA), are widely used as grinding aids and hydration accelerators in Portland cement systems [23,24,25]. Among these, TEA and TIPA have been shown to accelerate the hydration of aluminates and ferrites through chelation and to influence the crystallization behavior of hydration products [26,27,28,29]. DEIPA, in particular, exhibits dual functionality as both a grinding aid and a hydration promoter. It has been demonstrated to reduce particle agglomeration, improve powder fluidity, and enhance early-age strength development by promoting the hydration of C_3_A and C_4_AF phases and the formation of microcrystalline Ca(OH)_2_ [30,31,32]. Recent studies have further shown that alkanolamines can significantly improve the hydration activity and microstructure of BOFS–cement blends by increasing the solubility of mineral phases and modifying the morphology of C-S-H gel. For example, Yang et al. [25] reported that four types of alkanolamines (TEA, TIPA, DEIPA, MDEA) had the potential to decrease the Ca(OH)_2_ content, porosity, and change the C-S-H morphology in BOFS-cement systems. Their results found that compressive strength developed more with TEA and DEIPA than with TIPA and MDEA. Furthermore, Wang et al. [23] observed that these four alkanolamines could increase the elemental concentrations of [Ca], [Al], [Fe] and [Si] in the liquid phase of BOFS through chelation, due to an increase in the dissolution of the C_3_S, C_2_S and C_4_AF mineral phases.

Ethanol-diisopropanolamine (EDIPA) is a relatively new type of alkanolamine that has received limited attention in cementitious systems. Its unique molecular structure, containing both primary and secondary hydroxyl groups, offers enhanced flexibility for complexation with multivalent cations. These characteristics suggest that EDIPA may serve a dual function by improving grinding efficiency and promoting chemical activation through mechanochemical pathways. However, its effects on the grinding behavior, hydration kinetics, microstructural evolution, and mechanical performance of BOFS remain largely unexplored.

In this study, EDIPA was employed as a multifunctional additive, acting simultaneously as a grinding aid and a hydration accelerator to enhance the reactivity of BOFS. A comprehensive experimental program was carried out to evaluate the effects of different EDIPA dosages on the grinding efficiency, hydration behavior, and mechanical performance of BOFS pastes. The primary objective of this research is to elucidate the dual function of EDIPA in improving BOFS grindability and promoting its hydration kinetics. In addition, the research results provide mechanistic insights into the complexation of EDIPA and its impact on the formation and morphology of hydration products.

## 2. Experimental Section

### 2.1. Raw Materials

**Figure 1:**
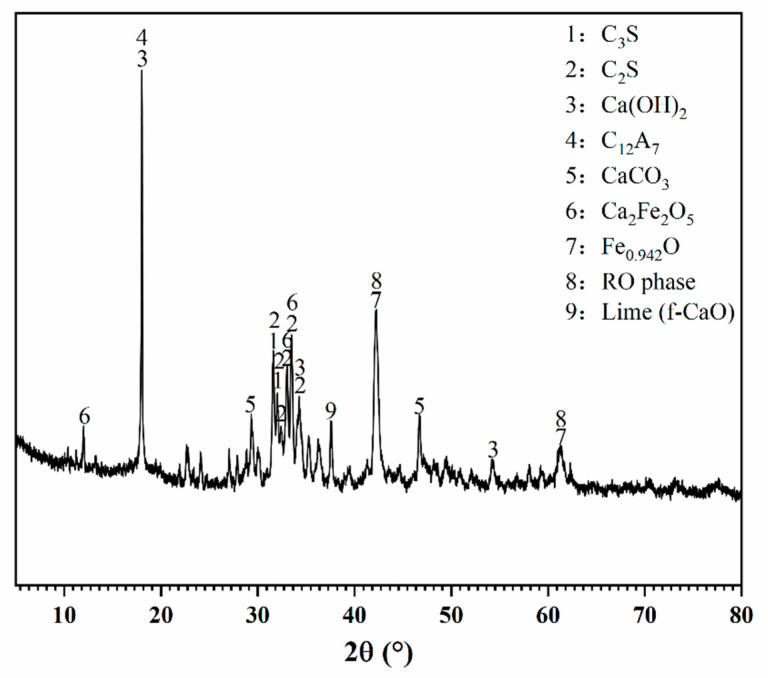
XRD pattern of BOFS.

**Figure 2:**
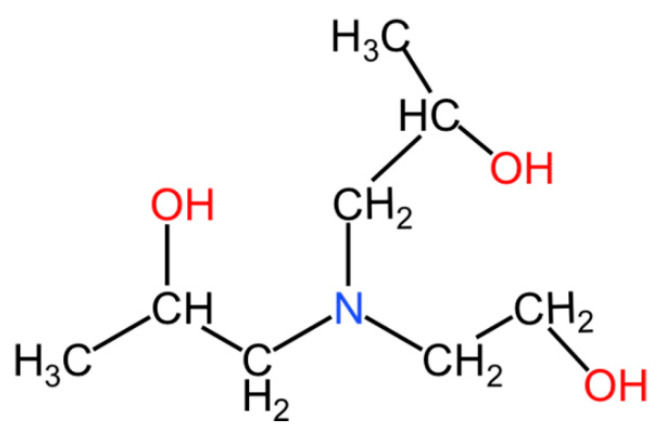
The molecular structure of EDIPA.

The BOFS used in this study was obtained from DaSteel Group Co., Ltd. (Dazhou, China). Its chemical and mineral compositions are presented in Table 1 and Figure 1. The EDIPA, with a purity of 85%, was supplied by Hongbaoli Co., Ltd. (Nanjing, China); its molecular structure is shown in Figure 2. The naming of samples follows the pattern ‘E’ + ‘dosage’. For example, E-0.02% refers to the sample with 0.02% of EDIPA.

### 2.2. Test Methods

#### 2.2.1. Particle Size Test

The BOFS coarse particles were dried at 105 °C until the moisture content was reduced to below 1%. Subsequently, the particles were crushed to a size smaller than 3 mm using a jaw crusher. During the grinding process, the fine BOFS particles were first ground for 20 min using a cement test ball mill (SM Φ500 × 500). EDIPA was then added to the BOFS coarse powder and co-ground for 30 min in a planetary ball mill. The EDIPA was added at dosages of 0.02%, 0.05%, 0.08%, and 0.1% by weight of BOFS. A control group without EDIPA was also prepared. The selected EDIPA dosage range (0.02–0.1 wt.%) was determined based on preliminary screening tests, which revealed enhanced performance within this interval. This range is also consistent with reported effective concentrations for alkanolamines in cementitious systems, typically falling between 0.01–0.1 wt.% of the binder mass, as supported by previous studies. The resulting ball-milled BOFS powder was used for subsequent testing. The specific surface areas of ground BOFS were measured using the Blaine method, following the Chinese standard GB/T 8074-2008 [33]. The sieve residue of BOFS at 30 μm, 45 μm, and 80 μm was determined in accordance with the Chinese standard GB/T 1345-2005 [34]. Particle size distribution was determined using laser diffraction with a Mastersizer 2000 analyzer (Malvern Instruments Ltd., Malvern, UK). The angle of repose was measured according to the standard GB 11986-1989 [35], using the fixed-height funnel method. A FT-104B angle-of-repose tester was employed for the measurement.

#### 2.2.2. Performance Test of BOFS Paste

##### Fluidity and Setting Time Tests

According to the Chinese standard GB/T 1346-2024 [36], the water-to-powder ratio for the BOFS paste was fixed at 0.22 to achieve a standard consistency. The initial and final setting times of the BOFS pastes, incorporating various dosages of EDIPA and 1.2% superplasticizer, were measured using a Vicat apparatus. The fluidity of the fresh paste was evaluated using a frustum-shaped mold. After the initial fluidity was recorded, the paste remaining in the container was stirred again after 30 min; the fluidity was measured a second time to determine the 30 min retention value.

##### Compressive Strength Test

According to the Chinese standard GB/T 17671-2021 [37], the fresh BOFS paste was cast into steel molds (20 mm × 20 mm × 20 mm) and demolded after 24 h. Subsequently, all specimens were cured in a controlled environment at 20 ± 1 °C and relative humidity of 95 ± 5% for the specified durations. These curing conditions were maintained consistently throughout the testing to ensure result comparability. Subsequently, all specimens were cured in air at 20 ± 1 °C and a relative humidity of 95 ± 5% prior to compressive strength testing. The effects of EDIPA additions on the compressive strength development of the hardened BOFS pastes were evaluated under a loading rate of 0.5 kN/s. Each strength measurement was repeated six times; the average values with standard deviations are reported to ensure reproducibility.

#### 2.2.3. Hydration Process Test of BOFS Paste

##### Hydration Heat Test

The early heat evolution of the BOFS paste containing different dosages of EDIPA was measured using an isothermal calorimeter (TA Instruments, New Castle, DE, USA). In this study, the liquid-to-solid mass ratio was maintained at 0.3, and the heat release profiles during the first 72 h of hydration were recorded at a constant temperature of 20 °C.

##### Paste Solution Conductivity Test

The ability of different EDIPA dosages to dissolve the mineral phases in BOFS was evaluated by measuring the electrical conductivity of the paste solutions. Deionized water was used as the dispersion medium and the paste solutions were prepared by mixing BOFS powder with water at a mass ratio of 1:6. A magnetic stirrer was operated at a speed of 450 rpm during the mixing process. Changes in conductivity were recorded every 10 min during the first hour, and subsequently every 30 min after one hour of stirring. Conductivity measurements were performed using a DDS-11A conductivity meter (Shanghai INESA Scientific Instrument Co., Ltd., Shanghai, China).

##### Element Concentrations Test

The dissolution of Ca, Al, and Fe ions from the BOFS paste in 0.05 mol/L NaOH solution was analyzed using inductively coupled plasma–optical emission spectrometry (ICP–OES). The experimental procedure was as follows: a water-to-solid ratio of 4:1 was set and the BOFS powder was dispersed in the sodium hydroxide solution. EDIPA was added at concentrations of 1 g/L and 2 g/L, with a blank sample prepared for comparison. The mixtures were stirred using a magnetic stirrer, then centrifuged at 10,000 rpm for 10 min. The supernatant was filtered through a 0.22 μm membrane. The elemental composition of the solutions was determined by ICP–OES.

#### 2.2.4. Hydration Products Test of BOFS Paste

##### X-Ray Diffraction Test

To identify the mineralogical composition of the hardened BOFS pastes, X-ray diffraction (XRD) analysis was conducted using a D/max-2500 diffractometer (Rigaku, Tokyo, Japan) equipped with Cu Kα radiation operated at 40 kV and 100 mA. The samples for XRD testing were prepared by grinding the reaction-terminated BOFS paste into fine powder. The diffraction patterns were collected over a 2θ range of 5° to 70° at a scanning rate of 10°/min.

##### TG/DTG Test

Thermogravimetric and differential thermal analyses (TG/DTG) were performed using an STA409C instrument (NETZSCH, Selb, Germany) under a nitrogen atmosphere. The samples were heated from room temperature to 950 °C at a rate of 10 °C/min. Prior to testing, all samples were thoroughly dried at 45 °C for 48 h and passed through a 75 μm sieve.

#### 2.2.5. Microstructural Test of BOFS Paste

##### Mercury Intrusion Porosimeter Test

The total porosity and pore size distribution of the cured BOFS pastes were characterized using a mercury intrusion porosimeter (Quantachrome PoreMaster GT-60, Boynton Beach, FL, USA). After 28 d of curing, the hardened pastes were crushed into pieces measuring 3–5 mm and immediately immersed in anhydrous ethanol to terminate hydration. The ethanol-treated samples were then dried at 45 °C for 48 h prior to testing.

##### Scanning Electron Microscopy Test

Specimens obtained from BOFS paste cured for 28 d were sectioned and immersed in anhydrous ethanol to terminate hydration, then dried at 45 °C until a constant weight was achieved. The dried samples were subsequently impregnated with epoxy resin and polished using sandpaper and 1 μm diamond paste. Backscattered electron (BSE) imaging and elemental mapping were performed at an accelerating voltage of 10 kV. The micromorphology of the polished paste surfaces was observed using a ULTRA 55 field-emission scanning electron microscope (FESEM, ZEISS, Jena, Germany) in secondary electron mode.

### 2.3. Chelation Capacity of EDIPA

The influence of different concentrations of the EDIPA on the electrical conductivity of Ca^2+^, Fe^3+^, and Al^3+^ under alkaline conditions was analyzed using a conductivity meter. Ion solutions of Ca^2+^, Fe^3+^, and Al^3+^ at 0.1 mol/L were adjusted to a pH of approximately 12.6 by adding 0.5 mol/L NaOH solution. After filtering to remove precipitates, equal volumes of the solutions were taken. EDIPA solution was added at varying concentrations; a blank group with deionized water was prepared for comparison. Then, the electrical conductivity of the solutions was then measured.

### 2.4. Synthesis of Ca(OH)_2_

To investigate the effect of EDIPA on the formation of Ca(OH)_2_, Ca(OH)_2_ was synthesized in pure solutions (deionized water and EDIPA solution). Following the synthesis method [38], 100 mL of 0.8 mol/L NaOH solution and 100 mL of 0.4 mol/L CaCl_2_ solution were added separately into 200 mL of deionized water and 2 g/L EDIPA solution, respectively. The precipitation reaction was carried out under slow stirring at a constant feed rate over 60 min. After completion of the reaction, the precipitates were filtered and dried to obtain the Ca(OH)_2_ samples.

## 3. Results

### 3.1. Grinding Efficiency

**Figure 3:**
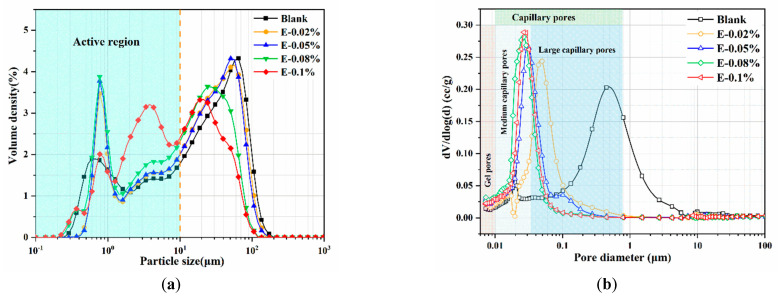
Pore structures of BOFS with different dosages of EDIPA: (**a**) particle size distribution; and (**b**) cumulative size distribution.

The grinding performance and particle size distribution of BOFS with different EDIPA dosages are shown in Table 2 and Figure 3. The addition of EDIPA significantly altered the particle size characteristics and distribution of BOFS, shifting the particle population from large-capillary pores toward capillary pores, resulting in pronounced particle size refinement. When the EDIPA dosage was increased to 0.1 wt.%, the D_50_ particle size decreased from 17.33 μm to 6.63 μm; the specific surface area increased from 345 m^2^/kg to 595 m^2^/kg; and the 30 μm sieve residue decreased from 35.4 wt.% to 3.9 wt.%. However, further increases in EDIPA dosage resulted in only marginal improvements in grinding efficiency. This diminishing return is likely due to oversaturation of the particle surfaces, where excessive adsorption of EDIPA may hinder further dispersion or even lead to steric stabilization effects. The observed reduction in particle size aligns with previous studies on alkanolamine-based grinding aids. This enhanced grinding behavior contributes to the increased surface area and thus to the accelerated hydration behavior observed in the discussion sections.

**Figure 4:**
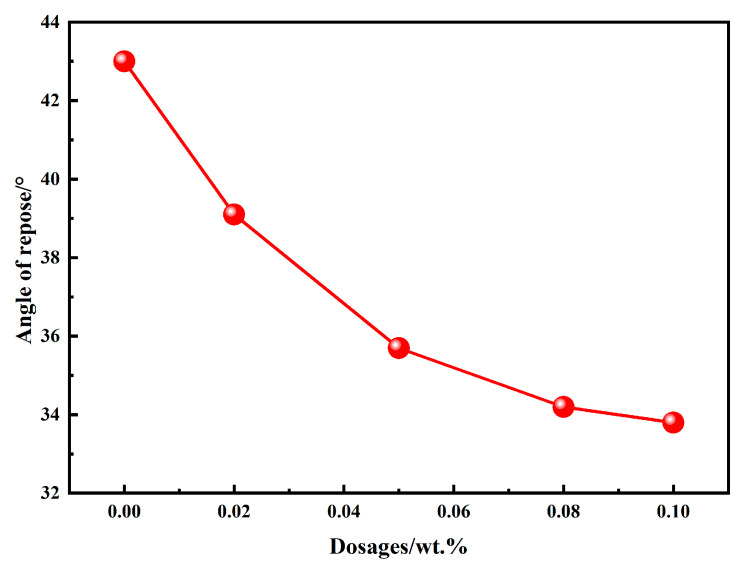
Angle of repose of BOFS with different dosages of EDIPA.

The angle of repose is commonly used to evaluate the flowability of powdery materials; a smaller angle indicates better flowability. Figure 4 illustrates the angle of repose of BOFS powders ground with different types and dosages of chemical additives. It can be observed that the angle of repose of BOFS powders with 0.02 wt.%, 0.05 wt.%, 0.08 wt.%, and 0.1 wt.% EDIPA decreased by 9.07%, 16.90%, 20.46%, and 21.40%, respectively, compared with the control group. The results indicate that EDIPA significantly improves the flowability of BOFS powders by reducing their angle of repose. Moreover, the angle of repose tends to decrease progressively with increasing EDIPA content, although the rate of reduction becomes less pronounced at higher dosages.

### 3.2. Performance

#### 3.2.1. Fluidity and Setting Time

**Figure 5:**
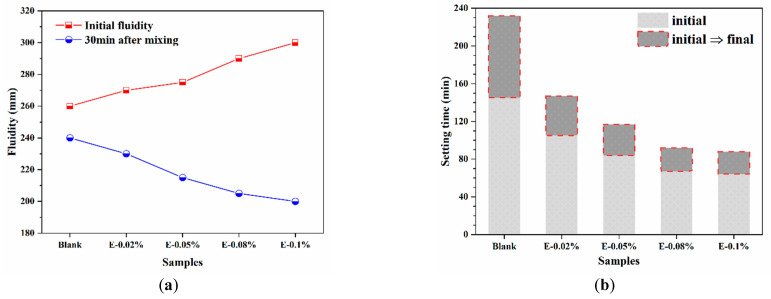
Working performance of BOFS pastes: (**a**) fluidity; and (**b**) setting time.

The working performance of BOFS pastes are shown in Figure 5. As shown in Figure 5a, the initial fluidity of the BOFS paste increased progressively with the addition of EDIPA. This improvement was attributed to the interaction between the hydroxyl groups on the side chains of EDIPA and water molecules, which formed a hydration film that enhanced particle dispersion and reduced interparticle attraction, thereby improving fluidity. In addition, this enhancement was also associated with the decrease in the angle of repose, which reflected improved particle morphology and surface smoothness, further facilitating better flowability. However, 30 min after mixing, the fluidity of all EDIPA-containing pastes became lower than that of the blank sample and decreased further with increasing EDIPA dosages. This trend suggests that EDIPA accelerated the dissolution of mineral phases in BOFS, consuming a significant amount of free water and releasing more heat, which in turn promoted early hydration reactions and reduced paste fluidity over time.

In addition to fluidity, the setting time is another critical parameter for material applications. As shown in Figure 5b, EDIPA markedly influenced the setting time of the BOFS paste. The initial and final setting times of the blank sample were 145 min and 232 min, respectively. With the incorporation of EDIPA, the setting time was significantly shortened. Notably, the E-0.08% sample exhibited initial and final setting times of 67 min and 92 min, which were 78 min and 140 min shorter than those of the blank, respectively.

#### 3.2.2. Compressive Strength

**Figure 6:**
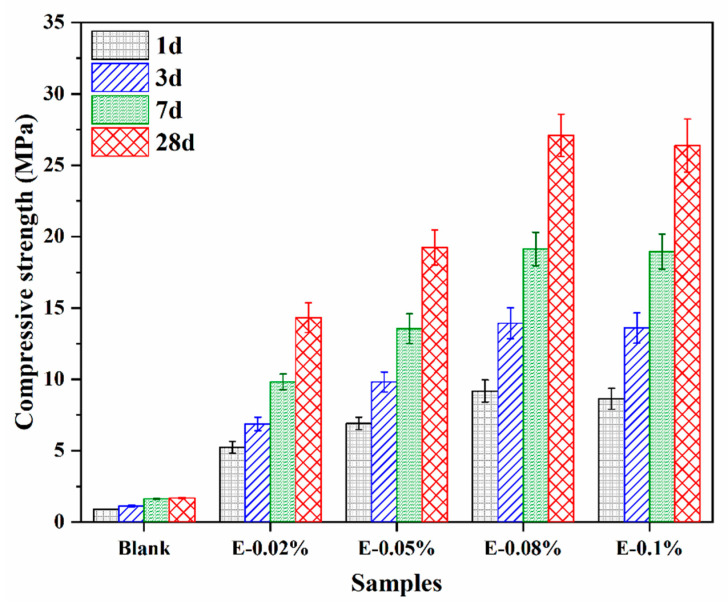
Compressive strength development of BOFS pastes containing various dosages of EDIPA.

To better understand the effect of EDIPA on the development of mechanical properties in BOFS pastes, the compressive strengths of hardened pastes at 1 d, 3 d, 7 d, and 28 d were measured, as shown in Figure 6. The blank group without EDIPA exhibited significantly lower compressive strengths at all ages compared to the EDIPA-containing pastes. Moreover, the strength development in the blank group was relatively slow, reaching only 1.69 MPa at 28 d. In contrast, the addition of EDIPA markedly enhanced the compressive strength of the BOFS pastes, with strength increasing rapidly over time. For example, the E-0.08% paste achieved compressive strengths of 9.18 MPa, 13.94 MPa, 19.14 MPa, and 27.09 MPa at 1 d, 3 d, 7 d, and 28 d, respectively, all of which were significantly higher than those of the blank at corresponding ages. It is noticeable that there was no significant improvement in compressive strength with the continued addition of EDIPA.

### 3.3. Hydration Process

#### 3.3.1. Hydration Heat

**Figure 7:**
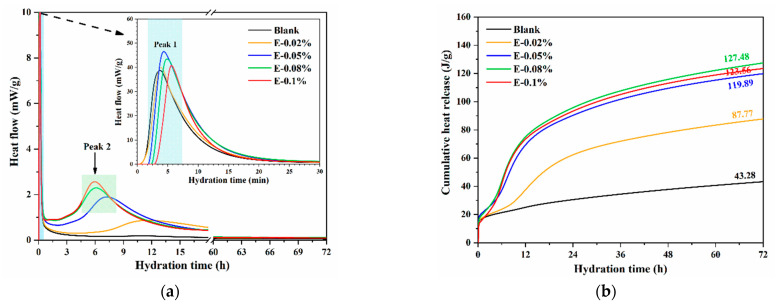
Hydration heat of BOFS pastes with different dosages of EDIPA: (**a**) heat flow; and (**b**) heat release.

The dissolution reaction rate of BOFS was evaluated by measuring the hydration heat. In this study, the effect of various EDIPA dosages on the hydration heat of BOFS was investigated. As shown in Figure 7a, the initial exothermic peak within the first 10 min corresponds to the rapid dissolution of reactive BOFS components, particularly C_2_F and C_12_A_7_, releasing Ca^2+^ and Al^3+^ into the solution. The subsequent broad peak, which is intensified by the addition of EDIPA, is associated with the formation of hydration products such as C-S-H and Mc phases. All BOFS pastes containing EDIPA exhibited higher initial exothermic peaks compared to the blank sample, indicating that EDIPA accelerated the dissolution of the primary mineral components in BOFS. Notably, the highest initial dissolution peak was observed in the paste with 0.05 wt.% EDIPA. This was likely due to the rapid formation of chelates between EDIPA and the dissolved mineral ions, along with EDIPA adsorption onto the surface of BOFS particles. When the EDIPA dosage was too high, this adsorption temporarily inhibited further dissolution. However, this inhibition diminished as hydration progressed, which is consistent with findings reported in previous studies [39,40]. Following the initial stage, the hydration heat flow of the blank sample gradually declined, whereas the pastes with EDIPA showed a secondary, pronounced exothermic peak between 5–15 h. The timing of this peak shifted earlier, and the magnitude increased, with higher EDIPA dosages.

Additionally, as shown in Figure 7b, the cumulative heat release over 72 h increased in the presence of EDIPA. The maximum cumulative heat was achieved at an EDIPA dosage of 0.08 wt.%, which was consistent with the results from the solution conductivity tests. Since the properties of hardened BOFS pastes are largely governed by the extent of hydration, these results suggest that EDIPA effectively activated BOFS, thereby enhancing compressive strength. Although the calorimetric analysis focused on the first 72 h, complementary characterizations, such as XRD, TG, MIP, and SEM, were conducted at 28 d.

#### 3.3.2. Conductivity of BOFS Paste

**Figure 8:**
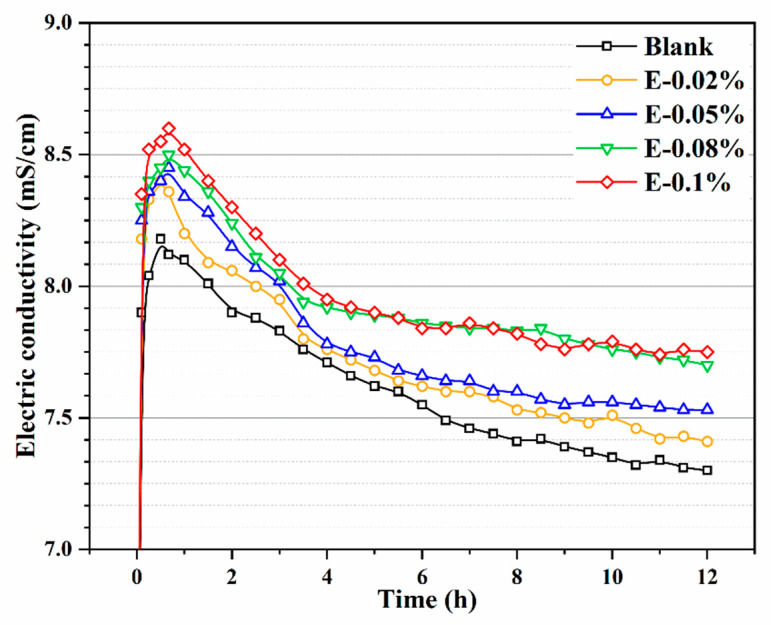
Electric conductivity of BOFS pastes with different dosages of EDIPA.

The conductivity of BOFS paste reflects its ionic concentration [41], which is considered a critical parameter for evaluating the ability of EDIPA to enhance BOFS dissolution. As shown in Figure 8, the conductivity profiles of BOFS paste systems with different EDIPA concentrations, along with a blank group, were recorded. In general, the ionic conductivity of the BOFS paste initially increased rapidly and then gradually declined as hydration progressed. This behavior was attributed to the dissolution of BOFS mineral phases in deionized water, which released a large number of metal ions and led to a sharp increase in conductivity at an early stage. At around 45 min, the ionic concentration approached saturation and the conductivity reached its peak. Due to prior grinding with EDIPA, the additive was well-dispersed in the paste and facilitated the early dissolution process. Simultaneously, the alkaline environment generated by BOFS hydration promoted the dissolution of Ca^2+^, Fe^3+^, and Al^3+^. The EDIPA exhibited strong chelating interactions with Fe^3+^, and Al^3+^, forming EDIPA-Fe^3^ and EDIPA-Al^3+^ complexes. These complexes further enhanced the dissolution of calcium ferrite and calcium aluminate phases in BOFS [23,40]. As the EDIPA dosage increased, both the peak and the stabilized conductivity values rose, indicating an improved dissolution capacity. However, the conductivity profiles of E-0.08% and E-0.1% eventually converged as hydration continued, suggesting that beyond a certain dosage, the effect of EDIPA on conductivity enhancement became less pronounced.

#### 3.3.3. Element Concentrations During BOFS Hydration

**Figure 9:**
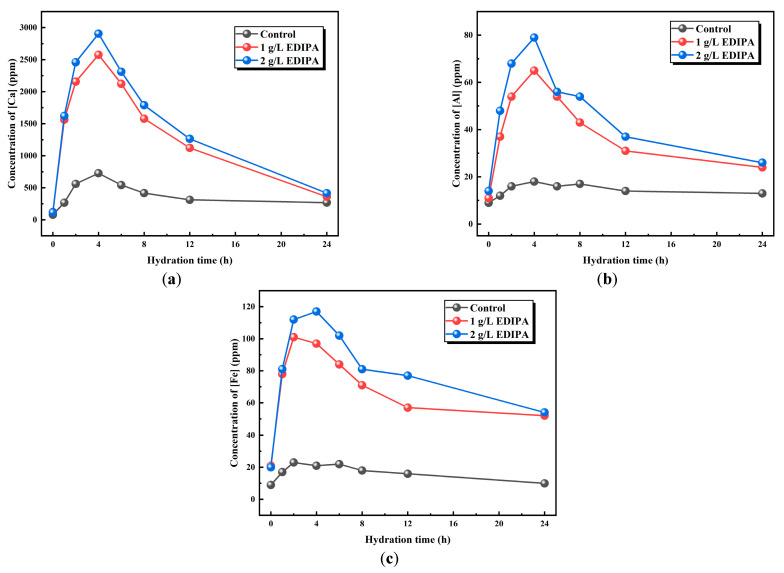
Effects of EDIPA on the element concentrations of: (**a**) [Ca]; (**b**) [Al]; and (**c**) [Fe] in BOFS paste.

Since Ca, Al, and Fe are the major elements in BOFS powder, the effect of EDIPA on the dissolution of these elements was preliminarily analyzed using ICP–OES. As shown in Figure 9, the concentrations of Ca^2+^, Fe^3+^, and Al^3+^ in the liquid phase were measured at various time intervals to evaluate the dissolution behavior. It was observed that the concentrations of these metal ions initially increased and then decreased as hydration progressed. This trend was attributed to the rapid dissolution of BOFS and the continuous precipitation of hydration products during the early hydration stage. In the later stages, with ongoing mineral dissolution and ion saturation in solution, precipitation of the hydration products became dominant, resulting in a decline in ion concentrations. This behavior was consistent with the conductivity trends shown earlier. Notably, the peak concentrations of Ca^2+^, Fe^3+^, and Al^3+^ occurred at approximately 4 h, which corresponded well with the maximum heat release observed in the hydration calorimetry tests. A comparative analysis revealed that the presence of EDIPA significantly enhanced the dissolution of all three ions, especially Fe^3+^, and Al^3+^. Furthermore, as the EDIPA dosage increased, its chelating effect on metal ions became more pronounced, promoting further dissolution from the BOFS.

### 3.4. Mineralogy

#### 3.4.1. X-Ray Diffraction Analysis

**Figure 10:**
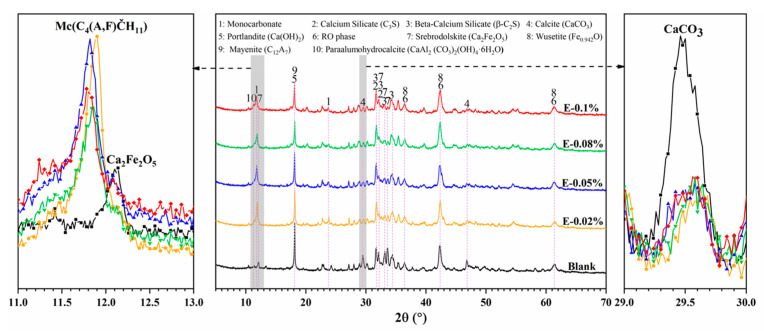
XRD pattern of BOFS pastes with different dosages of EDIPA at 28 d.

The XRD patterns of BOFS pastes with various EDIPA contents after 28 d of curing are presented in Figure 10. The main crystalline phases identified in the samples included Ca(OH)_2_, C_2_F, CaCO_3_, C_3_S, C_2_S and RO phases. Notably, diffraction peaks in the range of 11.6–11.9° (2θ), corresponding to monocarbonate (Mc), were observed only in the EDIPA-containing pastes and were absent in the blank group. CaCO_3_ was consistently detected at around 29–30° (2θ) in all samples. However, its diffraction intensity decreased with increasing EDIPA dosages. Furthermore, EDIPA altered the peak intensities of C_12_A_7_ (notably at 18.12°) and C_2_F (notably at 12.20°). This reduction may be attributed to the enhanced hydration of C_12_A_7_ and C_2_F facilitated by EDIPA, which subsequently reacted with CaCO_3_ to form Mc [42], as shown in Formulas (1) and (2):

(1) $$C_{12}A_{7}{\;+\;4\mathrm{CaCO}}_{3}{\;+53H}_{2}O\;\to {\;4C}_{4}{AČH}_{11}+\;3\mathrm{Al}{\left(\mathrm{OH}\right)}_{3}$$

(2) $$C_{2}{F\;+\;2\mathrm{CaCO}}_{3}{\;+\;25H}_{2}O\;\to {\;2C}_{4}{FČH}_{11}{\;+\;3\mathrm{Fe}(\mathrm{OH})}_{3}$$

In addition, the diffraction peaks of C_3_S and C_2_S were significantly weaker in the EDIPA-modified pastes compared to the blank, suggesting that EDIPA promoted the hydration of these mineral phases to varying degrees [43].

#### 3.4.2. TG/DTG Analysis

**Figure 11:**
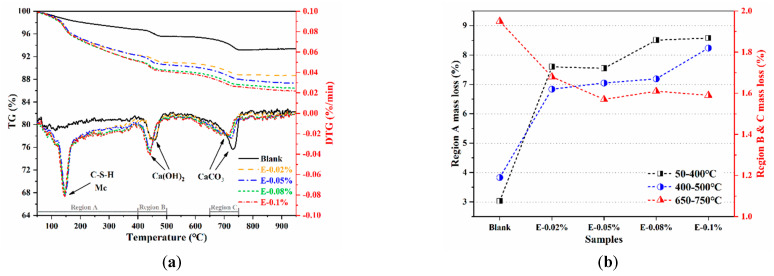
Thermogravimetry data for the BOFS pastes cured at 28 d: (**a**) TG/DTG; and (**b**) mass loss.

**Figure 12:**
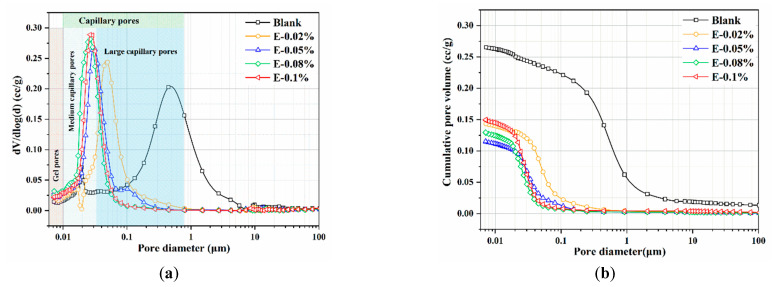
Pore structures of the BOFS pastes: (**a**) pore size distribution; and (**b**) cumulative pore volume.

To further investigate the activation effect of EDIPA on the BOFS pastes, the phase composition of the hydrated samples was analyzed using TG/DTG. In Figure 11a, the TG and DTG curves of the BOFS pastes cured for 28 d are presented. The first endothermic peak, observed at approximately 145 °C, was mainly attributed to the dehydration of C-S-H and Mc phases [44]. Notably, this peak was absent in the blank paste but became more pronounced with the incorporation of EDIPA, indicating the enhanced formation of C-S-H and Mc in the hydrated matrix. All samples also exhibited two additional endothermic peaks, located at approximately 400 °C and 750 °C, corresponding to the dehydration of Ca(OH)_2_ and the decarbonation of CaCO_3_, respectively [45]. The influence of EDIPA on BOFS hydration was further assessed by analyzing the mass loss in specific temperature regions, as shown in Figure 12b. These regions are defined as: Region A (50–400 °C), Region B (400–500 °C), and Region C (650–750 °C) [46]. In Region A, the mass loss increased from 3.03% in the blank paste to 8.51% in the paste with 0.08 wt.% EDIPA. This increase was attributed to the release of chemically bound water from hydration products, indicating that EDIPA promoted the hydration of C_3_S, C_2_S, C_2_F, and C_12_A_7_ phases in the BOFS [47]. The resulting enhancement in the formation of C-S-H and Mc contributed to improved mechanical properties.

In Region B, associated with Ca(OH)_2_ decomposition, mass loss also increased with the addition of EDIPA. However, this was in contrast to the XRD results, which showed a reduced Ca(OH)_2_ diffraction intensity in the EDIPA-containing samples. This discrepancy may be explained by EDIPA interfering with Ca(OH)_2_ crystal growth, leading to smaller or poorly crystalline Ca(OH)_2_ structures that were less detectable by XRD.

In Region C, the decarbonation peak of CaCO_3_ was significantly suppressed with increasing EDIPA content. This was likely due to the consumption of CaCO_3_ during the formation of Mc via the reaction of CaCO_3_ with hydrated C_12_A_7_/C_2_F phases. Moreover, the peak position in this region shifted to lower temperatures as the EDIPA dosage was increased. This shift was likely caused by lattice defects introduced by EDIPA during the mechanical grinding process, which reduced the binding energy of the calcite lattice and lowered its decomposition temperature.

### 3.5. Microstructure

#### 3.5.1. Pore Structure

In this study, the pore structure of BOFS pastes with different EDIPA contents was analyzed using MIP. As shown in Table 3, both the average pore diameter and total porosity of the BOFS pastes initially decreased and then increased with increases in EDIPA content, a trend that corresponded well with the observed compressive strength results. Among all samples, the BOFS paste with 0.08 wt.% EDIPA (E-0.08%) exhibited the most significant refinement in pore structure, with an average pore diameter of approximately 91.1 nm and a total porosity of 15.56%. The pore size distribution and cumulative porosity of the pastes cured for 28 days are presented in Figure 12. As shown in Figure 12a, the pore sizes are categorized into three types based on previous studies: gel pores (<10 nm), medium-capillary pores (10–50 nm), and large-capillary pores (>50 nm) [48,49]. The large-capillary pores, also referred to as harmful pores, are known to significantly compromise the mechanical strength of hardened pastes. With the incorporation of EDIPA, the proportion of large-capillary pores was reduced; many were converted into medium-capillary pores. Additionally, a slight increase in the proportion of gel pores was observed. From Figure 12b, it can be seen that the addition of EDIPA significantly decreased the cumulative pore volume. This was attributed to the increased generation of hydration products promoted by EDIPA, which filled the interconnected pores, altered pore connectivity, and ultimately led to a denser microstructure. The total porosity results show an approximate linear relationship with compressive strength. As the EDIPA dosage increased, the total porosity decreased, and the compressive strength increased.

#### 3.5.2. Morphology Analysis

**Figure 13:**
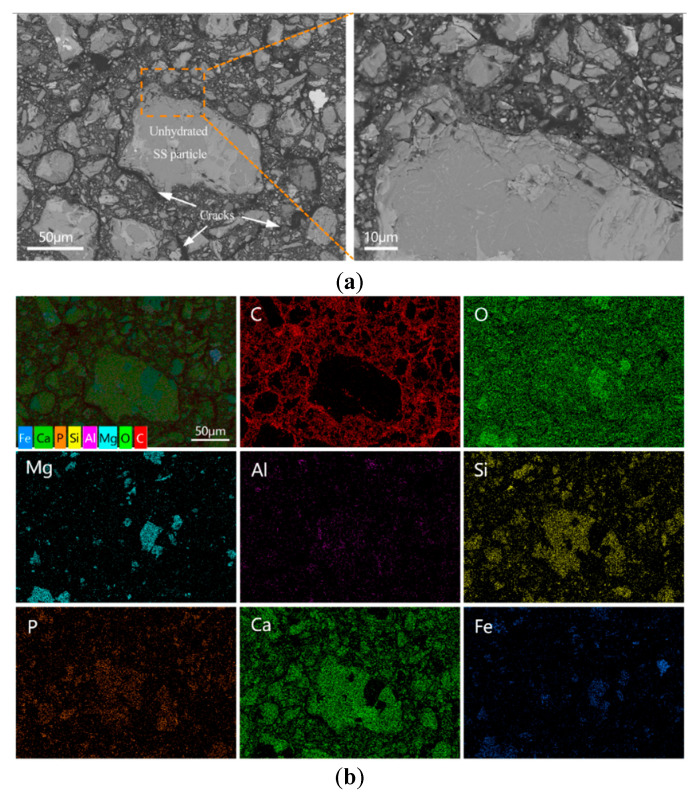
BOFS pastes without EDIPA cured at 28 d: (**a**) BSEM images; and (**b**) element mappings.

The BSE images of the polished fracture surfaces of the blank and E-0.08% samples after 28 d of curing, along with the corresponding elemental mapping results, are shown in Figure 13. In the blank sample (Figure 13a), numerous unreacted BOFS particles were clearly observed within the matrix. These particles exhibited a non-uniform size distribution and contained RO phases rich in Fe and Mg embedded in calcium silicate phases. The presence of such RO phases has been identified as one of the primary reasons for the poor grindability of BOFS particles. As shown in the elemental mapping image (Figure 13b), the main elements detected in a randomly selected area included C, Si, Ca, P, Mg, Fe, and O. The carbon signals primarily originated from the cured epoxy resin used for specimen preparation and were found concentrated in the cracks surrounding unhydrated BOFS particles. These interconnected cracks indicated a lack of structural continuity, which was a major contributor to the high porosity and low compressive strength observed in the blank sample.

**Figure 14:**
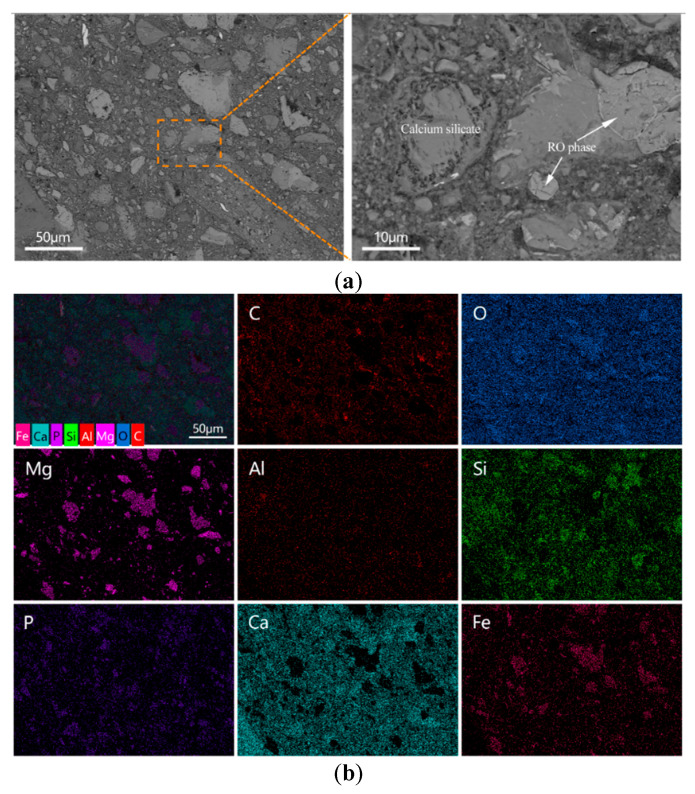
BOFS pastes with 0.08% EDIPA cured at 28 d: (**a**) BSEM images; and (**b**) element mappings.

In contrast, the microstructure of the E-0.08% sample (Figure 14a) appeared significantly more compact. Although some unreacted BOFS particles remained, they were mostly identified as RO phases [50]. In addition, more hydration products were observed to have formed around the BOFS particles, enhancing the interparticle bonding. The quantity of unreacted residues was noticeably reduced compared to the blank sample, suggesting that the addition of EDIPA promoted further hydration reactions and contributed to the consumption of BOFS particles.

**Figure 15:**
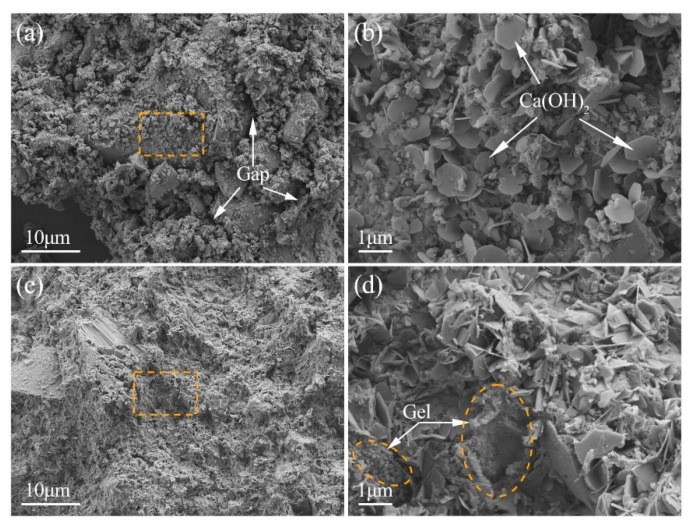
SEM images of BOFS pastes cured at 28 d: (**a**) control, with a lower magnification; (**b**) control, zoomed in; (**c**) E-0.08% at a lower magnification; and (**d**) E-0.08%, zoomed in.

The SEM images of the blank and E-0.08% samples after 28 d of curing are shown in Figure 15. As illustrated in Figure 15a, numerous unhydrated BOFS particles are loosely stacked, forming visible gaps throughout the hardened matrix. These voids are indicative of incomplete hydration and poor particle bonding. In the blank sample, Ca(OH)_2_ was found adhered to the surface of partially hydrated particles, exhibiting a characteristic hexagonal plate-like morphology, as shown in Figure 15b. In contrast, in the BOFS paste containing 0.08% EDIPA, the Ca(OH)_2_ has a more irregular and amorphous morphology. This transformation was attributed to the chelation effect between EDIPA and Ca^2+^ ions, which consequently lowered the supersaturation level of Ca(OH)_2_ and reduced its crystallinity [28,40]. Simultaneously, flocculent and agglomerated C-S-H gel structures can be seen in Figure 15c,d. These hydration products effectively filled larger capillary pores, refined the pore size distribution, and reduced the total porosity of the hardened matrix. As a result, the mechanical strength of the BOFS paste was significantly enhanced, consistent with the compressive strength and pore structure results discussed earlier.

## 4. Discussion

### 4.1. Effect of EDIPA on Performance of BOFS Paste

In this study, EDIPA was employed to mechanochemically activate BOFS. It was found that EDIPA significantly improved both the grindability and subsequent hydration performance of BOFS. The results revealed that the optimal dosage of EDIPA for enhancing both grindability and hydration performance was between 0.02 and 0.08 wt.%. Below this range, the effects on particle size and hydration kinetics were marginal; beyond 0.08 wt.%, no significant additional benefits were observed. Due to the high content of iron-bearing phases in BOFS, the material exhibited inherently poor grinding performance. The incorporation of EDIPA during the grinding process effectively enhanced grinding efficiency, increased the specific surface area, and reduced the angle of repose of the ground powder, thereby improving its flowability. These improvements were primarily attributed to the chelating and surface modification effects of EDIPA, which promoted particle dispersion and minimized agglomeration during the milling process.

Another critical limitation of BOFS in practical applications lies in its inherently low hydration reactivity. However, the mechanical strength tests of pastes prepared from ground BOFS revealed that the incorporation of EDIPA significantly enhanced compressive strength. Remarkably, the addition of only 0.02% EDIPA led to an over fivefold increase in the compressive strength of the BOFS paste. This enhancement could not be attributed to a single factor but rather resulted from the synergistic effects of multiple mechanisms. In the following sections, the mechanisms behind this improvement will be systematically analyzed from three perspectives: grinding behavior, hydration process, and microstructural evolution.

Compared with conventional alkanolamines, such as TEA, TIPA, and DEIPA, EDIPA exhibited comparable or superior grinding efficiency and hydration activation at significantly lower dosages (0.02 wt.%). From an economic perspective, EDIPA is commercially available at an approximate cost of USD 3000 per metric ton. Considering its low effective dosage of only 0.02 wt.%, the additive cost translates to less than USD 0.6 per ton of BOFS, highlighting its cost efficiency for large-scale applications.

### 4.2. Mechanism on Mechanical Strength Enhancement of EDIPA-Modified BOFS Paste

#### 4.2.1. Grinding Perspective

First, the enhancement in compressive strength of the BOFS pastes can be partially attributed to the improvement in grinding efficiency induced by EDIPA. By promoting particle dispersion and suppressing agglomeration during the grinding process, EDIPA significantly modified the particle size distribution of BOFS powders. As a consequence, the average particle size was markedly reduced, while the specific surface area increased notably, providing more reactive sites for subsequent hydration. This refinement in particle characteristics directly contributed to the acceleration of early-stage and mid-stage hydration reactions. According to previous studies, the particle size range that contributes most significantly to the reactivity of BOFS is generally below 10 μm [11,51]. The incorporation of EDIPA effectively increased the proportion of BOFS particles within this reactive range [30,52], which facilitated more complete and rapid hydration reactions. This enhancement led to the generation of a greater quantity of hydration products, contributing to the development of a denser and more refined microstructure.

#### 4.2.2. Hydration Perspective

From the perspective of the hydration process, the effect of EDIPA on BOFS paste was systematically evaluated through hydration heat evolution, electrical conductivity, and ion concentration measurements. The results demonstrated a consistent trend across the three characterization methods, validating each other and confirming that EDIPA significantly accelerated the hydration process of BOFS. Specifically, the addition of EDIPA led to a sharper initial exothermic peak in the hydration heat flow, a higher early-stage conductivity, and increased dissolution concentrations of key ions such as Ca^2+^, Al^3+^, and Fe^3+^.

**Figure 16:**
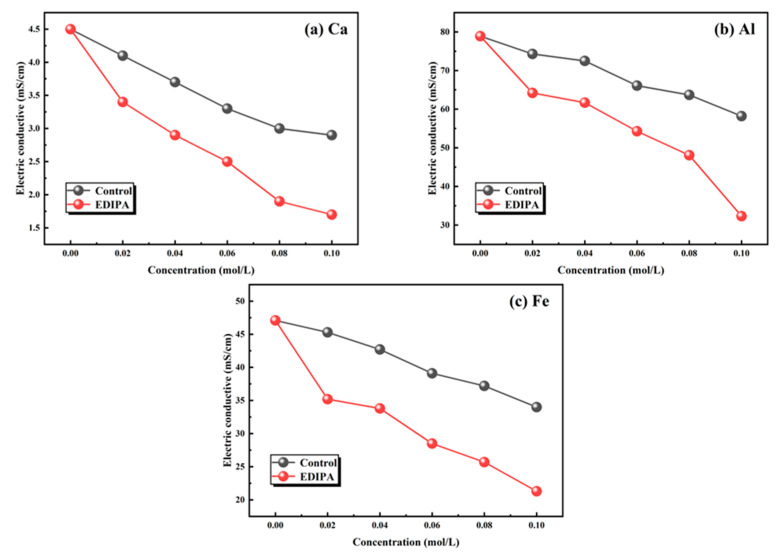
Chelation ability of EDIPA on: (**a**) [Ca]; (**b**) [Al]; and (**c**) [Fe].

The fundamental mechanism behind this activation effect lies in the chelating capability of EDIPA. In order to further verify this, the chelating capability of EDIPA with major metal ions in BOFS (Ca^2+^, Al^3+^, and Fe^3+^), was assessed. As shown in Figure 16, EDIPA exhibited strong chelation behavior with all three ions, as evidenced by the lower electrical conductivity values of their respective EDIPA-containing solutions compared to those in pure water. This decline in conductivity can be attributed to the formation of stable EDIPA-metal ion complexes, which reduced the mobility and availability of free ions in the solutions. Notably, since no precipitate was observed during the complexation reaction, the conductivity reduction was primarily a result of ionic mobility restriction rather than ion removal via precipitation. These findings suggest that EDIPA, by effectively binding with Ca^2+^, Al^3+^, and Fe^3+^ through its hydroxyl and amine functional groups, did not only promote the dissolution of mineral phases in BOFS.

The phase evolution trends identified by XRD were further corroborated by TG and ICP–OES results. As a consequence of the accelerated hydration induced by EDIPA, notable changes in the phase assemblage of the hardened pastes were observed. Most prominently, the emergence of Mc was detected in the EDIPA-doped systems, which was absent in the blank sample. In addition, the characteristic diffraction intensities of the C_12_A_7_ and C_2_F phases were markedly reduced with increasing EDIPA content, indicating that EDIPA significantly promoted their early-stage dissolution. This transformation in hydration products can be attributed to the strong chelating effect of EDIPA on Al^3+^ and Fe^3+^ ions. The complexation of these metal ions in the EDIPA enhanced the dissolution of c C_12_A_7_ and C_2_F phases, leading to a higher concentration of Al^3+^ and Fe^3+^ in the pore solution. These ions further reacted with CaCO_3_ to form Mc, which contributed to the densification of the microstructure by effectively filling capillary pores.

**Figure 17:**
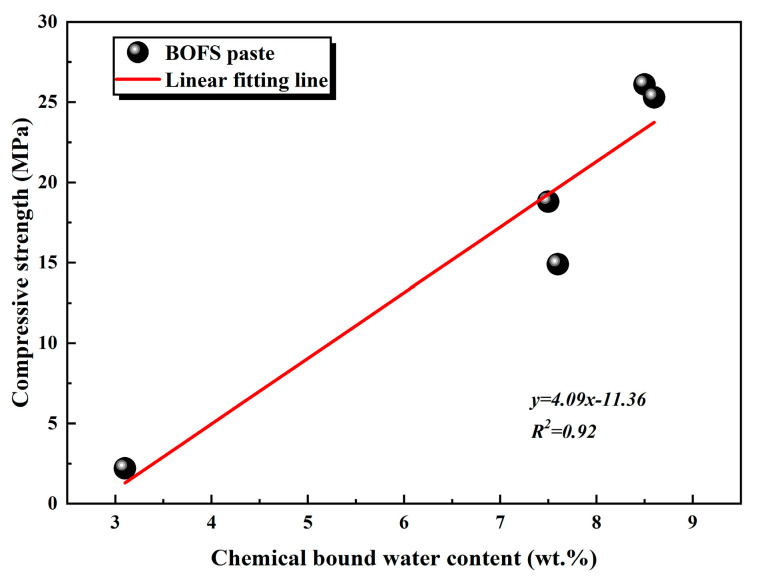
The relationship between chemical binding water content and compressive strength of BOFS paste at 28 d.

Furthermore, TG analysis (Figure 11) revealed an increase in the chemically bound water associated with the formation of C-S-H gel and Mc phases in the EDIPA-modified pastes. A quantitative evaluation of chemically bound water, based on the TG data, showed that EDIPA incorporation significantly enhanced the hydration degree of BOFS. To further verify the correlation between hydration product formation and mechanical performance, the chemically bound water content was linearly fitted against the measured compressive strength. As shown in Figure 17, a strong linear relationship was established, confirming the increase in C-S-H and Mc formation, which was a key factor contributing to the mechanical strength enhancement of the hardened BOFS paste.

#### 4.2.3. Microstructure Perspective

The microstructure of the BOFS pastes was comprehensively characterized using MIP, BSE, and SEM techniques. As illustrated in Figure 12, Figure 13 and Figure 14, the incorporation of EDIPA resulted in a significantly denser microstructure, as evidenced by reductions in both average pore diameter and total porosity. These enhancements were primarily attributed to the increased formation and filling effect of hydration products, which markedly increased with higher EDIPA dosages. Among the hydration products, C-S-H gel and Mc are recognized as the primary contributors to strength development in BOFS systems. By promoting the early dissolution of reactive mineral phases, EDIPA facilitated the enhanced precipitation of these phases, leading to notable pore refinement. This effect was clearly demonstrated by MIP, which showed a reduction in large-capillary pores and a shift towards a smaller gel and medium-capillary pores.

Another key factor contributing to microstructural densification was the modification of Ca(OH)_2_ crystal morphology. XRD analysis (Figure 10) revealed a decrease in Ca(OH)_2_ diffraction intensity with the addition of EDIPA, suggesting alterations in Ca(OH)_2_ crystallinity or particle size. Consistently, the SEM observations in this study (Figure 15) showed that the size of Ca(OH)_2_ crystals was significantly reduced upon the incorporation of EDIPA. Unlike the large, hexagonal plate-like Ca(OH)_2_ crystals observed in the blank samples, the EDIPA-modified pastes contained finer, more amorphous Ca(OH)_2_ morphologies. Previous studies have indicated that alkanolamines can influence the nucleation and growth of Ca(OH)_2_ crystals, resulting in smaller, irregularly shaped or amorphous forms [40,53].

**Figure 18:**
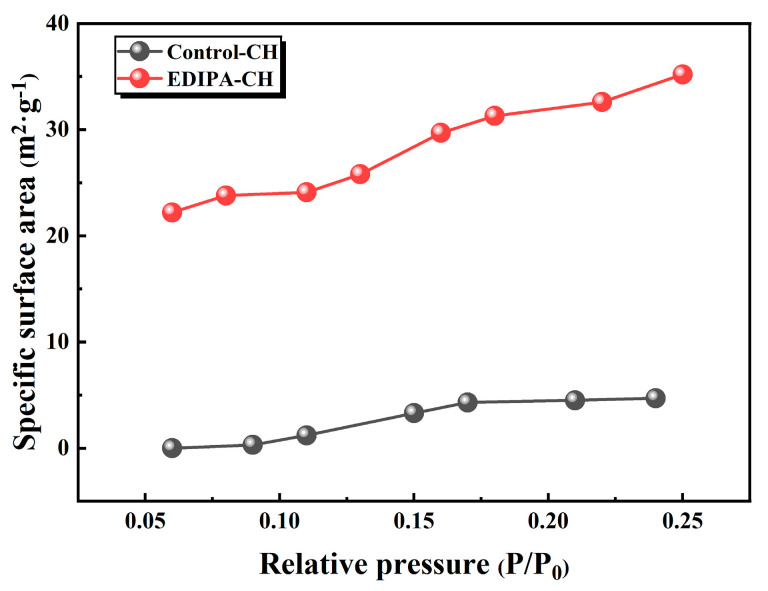
Specific surface area of Ca(OH)_2_ prepared in deionized water and EDIPA solutions.

To further investigate this phenomenon, Ca(OH)_2_ was synthetically precipitated in both deionized water and EDIPA solution and the specific surface area was measured via BET analysis. As shown in Figure 18, Ca(OH)_2_ synthesized in EDIPA exhibited a significantly higher specific surface area (35.2 m^2^/g) compared to Ca(OH)_2_ synthesized in deionized water (4.7 m^2^/g), indicating pronounced particle size refinement. This refinement bears important implications for microstructure development. According to a previous study [54], finely dispersed Ca(OH)_2_ crystals can embed within the C-S-H gel matrix or fill submicron pores within the cementitious network, thereby reducing pore volume. The observed improvement in compressive strength correlated well with the microstructural refinement confirmed by the SEM and MIP analyses. The formation of a denser matrix with reduced total porosity and refined Ca(OH)_2_ crystal morphology contributes significantly to the mechanical performance enhancement.

## 5. Conclusions

In this study, ethanol-diisopropanolamine (EDIPA) was employed as a multifunctional additive to enhance the grindability and hydration reactivity of basic oxygen furnace slag (BOFS) through a mechanochemical activation strategy. A comprehensive investigation was conducted to evaluate the effects of EDIPA on the compressive strength development, hydration mechanisms, and microstructural evolution of BOFS pastes. The key findings are summarized as follows:

(1) EDIPA significantly improved the grindability of BOFS and enhanced its mechanical performance. By reducing particle agglomeration and increasing the proportion of particles smaller than 10 μm, EDIPA refined the particle size distribution and reduced the angle of repose, thereby improving powder flowability. Notably, the incorporation of 0.08 wt% EDIPA led to remarkable compressive strength gains of 8.28 MPa (1 d), 12.82 MPa (3 d), 17.50 MPa (7 d), and 25.40 MPa (28 d) compared to those of the blank sample;
(2) Conductivity measurements demonstrated that EDIPA effectively complexed with Ca^2+^, Al^3+^, and Fe^3+^, especially Al^3+^, and Fe^3+^. This promoted the dissolution of reactive mineral phases, such as C_12_A_7_ and C_2_F, increasing the concentration of metal ions in the pore solution and accelerating subsequent hydration reactions;
(3) The addition of EDIPA shortened the time required to reach the peak of hydration heat release and the equilibrium point in conductivity, indicating a faster hydration rate. XRD and TG analyses confirmed the formation of Mc (C_4_(A,F)ČH_11_) in the EDIPA-modified systems, likely produced through the reaction of dissolved Al and Fe species with CaCO_3_. The increased formation of both C-S-H and Mc with higher EDIPA dosages contributed significantly to matrix densification and strength enhancement;
(4) BSE and SEM observations showed that EDIPA transformed Ca(OH)_2_ from large, hexagonal plates into smaller, amorphous particles. The Ca(OH)_2_ synthesized in the EDIPA solution exhibited a surface area of 35.2 m^2^/g, compared to 4.7 m^2^/g in deionized water, indicating substantial crystal refinement. This refinement, together with the enhanced formation of C-S-H and Mc, effectively reduced the average pore size and total porosity, leading to a denser microstructure and improved mechanical performance of the hardened paste.

## Figures and Tables

**Table 1:** Chemical compositions of BOFS measured by XRF (wt.%).

Component	CaO	Fe_2_O_3_	SiO_2_	P_2_O_5_	MgO	Al_2_O_3_	MnO	TiO_2_	LOI
	45.22	23.73	9.32	5.34	4.13	2.55	1.95	1.85	2.82

**Table 2:** The grinding effect of different dosages of EDIPA on BOFS.

EDIPA Dosage (wt.%)	Specific Surface Area (m^2^·kg^−1^)	Sieve Residue (wt.%)		
		30 μm	45 μm	80 μm
0	345	35.4	22.5	6.5
0.02	420	22.3	11.6	1.3
0.05	545	16.8	1.3	0.0
0.1	595	3.9	0.0	0.0
0.3	605	3.6	0.0	0.0

**Table 3:** Results of MIP analysis.

Sample	Blank	E-0.02%	E-0.05%	E-0.08%	E-0.1%
Total intruded volume (mL/g)	0.2175	0.1207	0.1244	0.1197	0.1169
Total surface area (m^2^/g)	7.45	11.91	15.47	18.62	16.14
Average pore diameter (nm)	116.8	40.5	32.2	25.7	28.9
Total porosity (%)	41.55	30.53	27.23	25.99	26.85

## Data Availability

The original contributions presented in this study are included in the article. Further inquiries can be directed to the corresponding author.
